# Pregnancy and the Postpartum Period in Women With Relapsing-Remitting Multiple Sclerosis Treated With Old and New Disease-Modifying Treatments: A Real-World Multicenter Experience

**DOI:** 10.3389/fneur.2020.00105

**Published:** 2020-02-25

**Authors:** Aurora Zanghì, Emanuele D'Amico, Graziella Callari, Clara Grazia Chisari, Giovanna Borriello, Luigi Maria Edoardo Grimaldi, Francesco Patti

**Affiliations:** ^1^Department “G. F. Ingrassia”, University of Catania, Catania, Italy; ^2^Institute Foundation “G. Giglio”, MS Center, Cefalù-Palermo, Italy; ^3^S. Andrea Hospital, Rome, Italy

**Keywords:** multiple sclerosis, pregnancy, NEDA 3, old DMTs, new DMTs

## Abstract

**Introduction:** Trends of disease activity during pregnancy, the postpartum period, and until 24 months from the delivery in the era of new drugs for the treatment of relapsing-remitting multiple sclerosis (RRMS) need to be investigated.

**Methods:** In this cross-sectional Italian multicenter study, women with RRMS were included; the disease-modifying treatment (DMT) at the time of conception included were: interferons, glatiramer acetate, teriflunomide, dimethyl fumarate, fingolimod, and natalizumab. The main outcome of the study was to determine the rate of relapse occurrence during pregnancy and the postpartum period in all women grouped for each DMT. The secondary outcome was to determine the overall disease activity assessed by NEDA 3 (relapse, disability level, and radiological activity) at 24 months from the date of delivery.

**Results:** Completed data were available for 81 pregnancies (in 74 women). Women on interferons and glatiramer had longer disease duration than women on dimethyl fumarate, fingolimod, and natalizumab (*p* < 0.05). Overall, we recorded 25 relapses during pregnancy (11 in 11 women) and the postpartum period (14 in 14 women). Natalizumab was the most commonly DMT in women (3) who experienced relapses during pregnancy. IFNs were the most commonly prescribed DMT in women (8) who experienced relapses during the postpartum period. At logistic regression analysis, specific treatment *per se* was not associated with relapse occurrence. No differences among the DMTs groups were recorded about NEDA 3 status at 24 months of follow-up.

**Conclusions:** In our population, there was no difference in terms of relapses occurrence, disability status, and the overall disease activity during a follow up of 24 months.

## Introduction

In women with the relapsing-remitting form of multiple sclerosis (RRMS), pregnancy still represents a challenge in terms of what to do before, during, and after such an event (1). Usually, women with RRMS and MS-physicians share a plan of pregnancy when patients become stabilized with absence of disease activity and are early in their disease course. Pregnancy has been associated with a 70% reduction in the annualized relapse rate (ARR) in the third trimester, compared to the year before pregnancy (2), but also with an increase in relapse frequency in the postpartum period (3). It is important to note that relapses occur most frequently in the 2–3 months postpartum (2–9).

The pathophysiologic pathways involved in the changes of MS activity during pregnancy and the postpartum period are still largely unknown. It is thought that estrogen and other sex hormones play a fundamental role in modulating T- and B-lymphocyte functions (10). Moreover, also the CD56 natural killer cells might play an active role in the reduction of disease activity during the third trimester (11). With the advent of new and potent—but less safe—disease-modifying treatments (DMTs) for RRMS, new issues in terms of management and pregnancy have arisen; of these, the timing of DMT wash-out pre-pregnancy, use during pregnancy, restart therapy after delivery, and the risk of disease activity rebound represent the most challenging. Literature provides data mainly for women who were on either interferon beta (IFN-β) or glatiramer acetate (GA) before conception (12–18), while few reports are disposable for the women on DMTs, which were released onto the market more recently (19–21).

Therefore, we aimed to describe the trends of MS activity during pregnancy and the postpartum period in an Italian multicenter population of women who received old (injectables) and new (oral and injectables) DMTs before conception.

## Methods

### Patients' Cohort

#### Database and Study Population

In this cross-sectional multicenter study, women with RRMS were identified from three large tertiary MS centers in Italy. The data entry used iMed^©^ software (iMed, Merck Serono SA; Geneva), and a rigorous quality assurance procedure was ensured regularly and locally by the treatment clinics using patient health records while coordinating with the iMed^©^ software data coordinators (22).

There were some key eligibility criteria: (i) women with a laboratory-supported or clinically established RRMS diagnosis (Poser or 2010 McDonald criteria) (23); and (ii) at least 12 months of follow-up after delivery.

There were some exclusion criteria: (a) women with others forms of MS than RRMS; and (b) women who were not on treatment at the time of conception. Ongoing pregnancies and abortions were also excluded.

The index window considered was from January 1st, 2005, to June 30th, 2017.

Each patient is usually followed prospectively with scheduled visits (at least one visits every 6 months).

The minimum dataset for all enrolled women included demographic data and clinical characteristics (age at inclusion, age at onset, disease duration, number of relapses and level of disability before and during the pregnancy and postpartum period (3 months from the delivery), duration of pregnancy dichotomized by trimesters, breastfeeding, prior/ongoing use of DMTs, therapeutic washout period, and the number of lesions at brain MRI).

Other general clinical data, such as body mass index (BMI), smoking status pre-, during, and post-pregnancy, and any comorbidities, were recorded at the time of enrollment; if any change occurred along the follow-up this was also recorded.

Data were extracted from the registry database on June 30th, 2018.

#### Procedures and Outcomes

Relapses were defined as new or recurrent neurologic symptoms not associated with fever or infection that lasted for at least 24 h. All relapses were treated with 1 g IV methyl prednisolone (IVMP) for 5 days.

Disability was scored using the Expanded Disability Status Scale (EDSS) administered by trained neurologists with an online Neurostatus certification (https://www.neurostatus.net/) who considered a EDSS evaluation for at least 30 days from any clinical relapse.

Brain MRI sequences were acquired with a 1.5 Tesla system. We collected the number of T2, T1, and T1 gadolinium (God+) lesions at the baseline (a scan performed within the 6 months prior the conception).

Then, we followed up the women through a brain MRI scan within the first trimester after delivery and then through an annual MRI scan.

Regarding treatment, changes in DMTs' use (including reasons for discontinuation/interruption) were noted. We considered old DMTs as the old injectables drugs: intramuscular IFN-β-1a, subcutaneous IFN-β-1a, subcutaneous IFN-β-1b, and GA. We considered the following as new DMTs: teriflunomide, dimethyl fumarate, fingolimod, and natalizumab. All DMTs were prescribed in accordance with the approved label instructions and the expected standards for good clinical practice.

To describe the presence of overall disease activity during the first 24 months of the postpartum period, we used the no evidence of disease activity (NEDA 3) score. NEDA 3 is a composite score obtained from three related measures of disease activity: (1) no evidence of relapses, (2) no EDSS score progression sustained for 6 or more months (1 EDSS step until the baseline score is EDSS 5.5 and 0.5 EDSS step if the baseline EDSS is >5.5), and (3) no MRI activity (new or newly enlarging lesions in T2 sequences and/or new God+ lesions at brain MRI) (24, 25).

### Study Endpoints

The main outcome of the study was to determine the rate of relapse occurrence during pregnancy and the postpartum period in women with RRMS treated with different DMTs at the time of conception.

Relations between any demographic and clinical factors and relapse occurrence during pregnancy and the postpartum period were assessed.

The secondary outcome was to determine the level of disease activity assessed by NEDA 3 at 24 months from the date of delivery.

### Data Analysis

Baseline characteristics were summarized as frequencies (%), mean with standard deviation, or median with range (min–max). Non-parametric tests (Mann–Whitney U-test) were utilized. Qualitative variables were analyzed with the Pearson χ^2^ and Fisher exact test.

Two different binary logistic regression models were built to binarily explore the effect of demographical and clinical variables on the probability of getting a relapse (expressed as dichotomic 0/1) during pregnancy and during the postpartum period, respectively.

For the event of relapse during pregnancy we investigated several variables: patient's age at inclusion (as a continuous variable), BMI (<19.5, 19.5–24, and >24), smoking status (as dichotomic 0/1), disease duration (as a continuous variable), DMT before pregnancy, length of the therapeutic washout period (as a continuous variable), number of relapses, and level of disability within 12 months before conception, number of T2, and T1 Gd+ brain MRI lesions within 12 months before conception (all as a continuous variable).

For the event of relapse during the postpartum period, we investigated several variables: relapses during pregnancy (as dichotomic 0/1), treatment continuation during pregnancy (as dichotomic 0/1), time to restart therapy after delivery (months, as continuous variable), and breastfeeding (as dichotomic 0/1).

The statistical software SPSS version 21.0 was used for all analyses (IBM SPSS Statistics 21, IBM^©^, Armonk, NY, USA).

### Standard Protocol Approval, Registration, and Patient Consent

A generic informed consent form was given to the patient before data entry to grant their approval before data entry. Any specific information about the study was discussed with the patient. The institutional ethics committee Catania 1 approved the study, and informed consent forms were obtained from all patients. Confidentiality and data protection were ensured in keeping with the recommendations of the declaration of Helsinki; written informed consent was obtained from every patient at first visit.

This study received no financial support for the design, data collection, data analysis, data interpretation, or the writing of it.

The corresponding author had full access to the entire database and had the final responsibility of submitting this manuscript.

## Results

**Figure 1:**
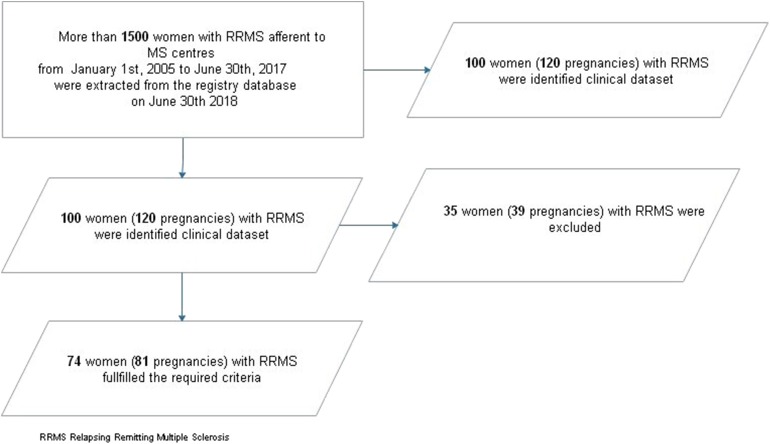
Patients' selection flow-chart. RRMS, relapsing-remitting multiple sclerosis.

A total of 120 pregnancies from the clinical datasets was identified. Eighty-one pregnancies (74 women) fulfilled the required criteria and were subsequently analyzed (see Figure 1).

Baseline demographical, clinical, and radiological characteristics are showed in Table 1.

**Table 1:** Baseline demographics and clinical characteristics of the studied cohort.

Variables^*^	
Mean age at inclusion, y	36.2 ± 5.9
Mean BMI	23.3 ± 3.5
Smoking pre-conception, *n* (%)	15 (18.5)
Mean age at delivery, y	30.1 ± 4.7
Mean disease duration, y	7.5 ± 7.5
EDSS score within the year before pregnancy, median (IQR)	2.0 (1.0–3.5)
N. of MRI T2 lesions in the year before conception	18 ± 17.1
N. of MRI T1 Gad+ lesions in the year before conception	0.7 ± 4.6

*n = 81 pregnancies, 74 women*.

* *Values are mean ± SD when otherwise specified. BMI, Body Mass Index; DMT, disease-modifying therapy; Gad+, Gadolineum; MRI, magnetic resonance imaging; y, years*.

Table 2 shows the demographical, clinical and radiological data according to the name of DMTs' used before conception. Women on IFNs and GA had longer disease duration than women on dimethylfumarate, fingolimod, and natalizumab (*p* < 0.05). Incidental pregnancy occurred in one woman who was prescribed teriflunomide, and an accelerated clearance procedure was carried out on pregnancy confirmation.

**Table 2:** Baseline characteristics according to DMT used.

Variables^*^	**IFN-β**	**GA**	**TNF**	**DMF**	**FYG**	**NTZ**
N. of pregnancies (n of women)	40 (37)	5 (3)	1 (1)	4 (4)	4 (4)	27 (25)
Mean age, y	37.2 ± 6.2	41 ± 3.5	39	35 ± 8.3	34.2 ± 4.9	35.8 ± 5.3
Age at delivery, y	29.7 ± 4.5	30.3 ± 2.7	37	32 ± 7	30 ± 5.1	33.4 ± 4
Disease duration, y	12.4 ± 5.5	15.9 ± 1.3	8	8.3 ± 4.8	11.8 ± 4.3	11.8 ± 4.6
Wash out period (months)	0.6 ± 0.7	2 ± 2	0	1.8 ± 0.9	3.2 ± 3.6	2.9 ± 0.6
N. of relapses (n. of women) 12 months before pregnancy	15 (15)	1 (1)	0	1 (1)	1 (1)	4 (3)
EDSS score, median (IQR) at conception	1 (1.0–2.0)	1.5 (1–2.0)	1.5 (1.0–2.0)	1.5 (1.0–2.0)	1.5 (1.0–2.0)	2 (1–2.5)
N. of pregnancies (n. of women) with MRI activity 12 months before pregnancy	2 (2)	0	0	0	2 (2)	3 (3)

* *Data are expressed as mean ± standard deviation when otherwise specified*.

*DMF, dimethylfumarate; DMTS, disease modifying therapy; EDSS, Expanded Disability Status Scale; FYG, fingolimod; GA, Glatiramer acetate; IFNs, interferons; MRI, Magnetic Resonance Imaging; n, number; NTZ, Natalizumab; TNF, teriflunomide; y, year*.

Women on natalizumab (of whom three continued the treatment until the end of the second trimester on the basis of a shared physician/women decision) had a higher EDSS score than women on IFNs within the 12 months before conception. The other variables showed no differences (Table 2).

Table 3 reports the trends of disease activity in terms of relapse occurrence during pregnancy and the postpartum period. Overall, we recorded 25 relapses during pregnancy and the postpartum period (in 24 women). The ARR pre-pregnancy was 0.40, while the ARRs during pregnancy and the postpartum period were 0.16 and 0.20, respectively.

**Table 3:** Characteristics of relapses during pregnancy and the postpartum period.

**Variables**	
**Relapses during pregnancy** **n (n. of women)**	11 (11)
IFNs	1 (1)
GA	0
TNF	0
DMF	2 (2)
FYG	2 (2)
NTZ	6 (6)
**Relapses during post-partum** **n (n. of women)**	14 (14)
IFNs	8 (8)
GA	0
TNF	0
DMF	0
FYG	2 (2)
NTZ	3 (3)

*N, number*.

Eleven relapses occurred during pregnancy. Most relapses were clustered around the first trimester (one at 8 weeks and six relapses at 12 weeks) and the third trimester (four relapses clustered from 28 to 34 weeks) of gestation. No relapses resulted in sustained residual disability.

Natalizumab was the most commonly used DMT in women who experienced relapses during pregnancy (see Table 3).

During the postpartum period, 14 relapses occurred. Most relapses were clustered around 4–8 weeks (11 relapses) and three around 10–12 weeks. IFNs was the most commonly prescribed DMT in women who experienced relapses during post-partum.

Two relapses resulted in sustained residual disability. One woman was treated with intramuscular IFN-β-1a; here the EDSS score before pregnancy was 1.5 and 12 months after delivery was 3.5, and no MRI load increase was observed.

The second women with residual disability had an EDSS score of 1.5 before pregnancy, and it moved to the value of 4.0 at 12 months after delivery. Here, MRI load increased (from 30 to 90 T2 MRI lesions).

In our cohort, 67 women had a natural delivery (labor was induced in two pregnancies after 41 weeks ± 5 days, and preterm birth between 34 and 37 weeks occurred in two pregnancies). Fourteen had a cesarean delivery. Thirty-nine patients breastfed, and the mean time to restart therapy was 1.6 ± 1.8 months.

The logistic regression models did not retain any variables. In detail, specific treatment *per se* was not associated with a relapse occurrence during pregnancy, and this was likely because of the low number of events once DMTs were dichotomized into specific variables.

NEDA 3 at 24 months data was recorded for all women. No differences among the DMTs groups were recorded (Table 4).

**Table 4:** NEDA 3 and its component distribution between the groups.

	**NEDA 3** **N. pregnancies** **(n. of women)**	**NEDA Relapse**	**NEDA EDSS**	**NEDA MRI**	p-value^*^
IFNs	30 (30)	30 (30)	36 (36)	36 (36)	ns
GA	3 (3)	3 (3)	3 (3)	3 (3)	ns
TNF	1	1	1	1	ns
DMF	4 (4)	4 (4)	4 (4)	4 (4)	ns
FYG	3 (3)	3 (3)	4 (4)	4 (4)	ns
NTZ	18 (18)	25 (22)	22 (22)	23 (22)	ns

* *via Fisher Exact test*.

No major fetal anomalies or congenital malformations were observed. Three newborns had a low birth weight, and one reported neonatal jaundice that resolved spontaneously.

## Discussion

Our multicenter experience confirms the literature data, which showed that pregnancy and the postpartum period appears to have no influence on the progression of disability in MS. The rate of relapse in our population during and before pregnancy was in accordance to previous meta-analysis data (4). Moreover, among the DMTs that we use, there was no difference in the impact on the rate of relapse, disability status, and the overall disease activity during a follow up of 24 months.

Our findings may raise questions regarding several practical points. Firstly, also women with active (in terms of relapse) MS history may plan their pregnancies, even if they are on high-efficacy therapies, such as natalizumab and fingolimod (in Italy both are licensed as second-line DMTs). It is described that continuing natalizumab during the first trimester could decrease the risk of relapses. Regarding fingolimod, it is suggested that the drug be withdrawn 2 months before pregnancy planning because fingolimod has been associated with teratogenic effects (26).

For teriflunomide and dimethyl fumarate the data are scarce. The former needs to be washed out with an elimination procedure, while a definitive washout period needs to be established for the latter. Some evidence, however, suggested about 2 weeks because of its short half-life (27). A similar issue was identified for alemtuzumab, which requires at least a 4-month washout period (27).

We did not find any specific role as predictors of relapse occurrence during pregnancy and the postpartum period for any of demographic and clinical investigated factors. It was recently found that longer washout treatment periods were linked to about 4-fold increase in the relapse occurrence during pregnancy (19); this was not the case in our cohort. Another study, analyzing data from 92 pregnancies in 83 women receiving natalizumab, showed that relapse rate during and after pregnancy was higher in women treated with natalizumab (*p* < 0.001) (28).

In the multivariable analysis, a longer natalizumab washout period was the only predictor of relapse occurrence during pregnancy (*p* = 0.001) (28). In a large registry study, performed on an international MS registry (MS Base) in which 893 pregnancies(674 women) were included, examining 3 period epochs (1967–2002; 2002–2006, and 2006–2010), any second-line or new DMTs were disposable (29). The women on DMTs were 39.0% in the 2-year period before conception (29). Here, the pre-conception ARR and DMT predicted early postpartum relapse in a multivariable model (29).

Two studies from Kuwait (DMT use rate 89.9%) and from Spain (DMT use rate 97.3%) showed an increase in the rate of relapses during pregnancy that was significantly higher in the group of women treated with natalizumab or fingolimod compared to the group of women treated with interferon beta or glatiramer acetate (5, 19, 30). Furthermore, in a Western Austria cohort, 387 pregnancies in 239 women with RRMS were analyzed; here, the risk of relapse during pregnancy and the postpartum period was predicted by the use of highly effective disease-DMTs pre-conception and by a prolonged washout period (6).

In the clinical practice, women with MS usually plan their pregnancies by sharing with MS physicians when the disease seems stable in terms of relapses and MRI activity; they and tend to consider pregnancy when they have low disability level. About DMTs used during gestation, most literature data are disposable for the old injectables, and, in 2017, pregnancy contraindication was removed from Copaxone 40 mg/ml three times a week (Teva Pharmaceuticals®) in Europe; more recently, in 2019, also Interferon Beta 1a at a dose of 44 mcg weekly (Merck-Serono®) was authorized to be used during pregnancy as clinically needed and while breastfeeding.

Obviously, the heterogeneity in terms of MS history (disease duration, relapses, and disability status) and in terms of different DMTs used might have influenced and affected our findings.

However, to establish a definitive interval between time of cessation of the DMTs' effect and the beginning of pregnancy has not been possible so far. We have no proper pharmacokinetics studies, and the serum concentration of any drug does not reflect its effects, which can be sustained by different metabolites. In the clinical practice, we based our decision on clinical or laboratory observations of the normalization of lymphopenia in fingolimod-treated women.

We did not find any role for breastfeeding in terms of impacting the MS course. It was described that breastfeeding may have protective effects in the postpartum period, but the data are conflicting with two recent studies showing the opposite (31–33).

Our study has some limitations. First, it has a retrospective design even if we referred to prospectively collected data. Second, we acknowledge the bias in saying that women who have milder disease were more likely to become pregnant, as described elsewhere (34). Overall, our data analyses may suffer from the fact that we were not able to perform subgroup analyses given the low number of women enrolled in the different DMTs groups.

However, our study is among the first studies conducted with pregnant women who were previously on second-line therapies, such as natalizumab, fingolimod, and dimethyl fumarate.

We have confirmed that pregnancy and postpartum periods did not negatively impact disease evolution; and we suggest that further discussion about pregnancy topics is a priority in MS management that should also be initiated as soon as possible after MS diagnosis.

We need randomized and prospective studies to gain more insight into the benefits and risks of the different DMTs and their timing regarding washout and restart therapy.

## Data Availability Statement

The datasets generated for this study are available on request to the corresponding author.

## Ethics Statement

This study was carried out in accordance with the recommendations of the Ethics Committee of Catania (Catania 1) with written informed consent from all subjects. All subjects gave written informed consent in accordance with the Declaration of Helsinki. The protocol was approved by the Ethics Committee of Catania (Catania 1) (No. 20/2018/PO).

## Author Contributions

ED'A and AZ contributed to data curation as well as the drafting, reviewing, and editing of the manuscript. GB, CC, and GC contributed to data curation. LG and FP contributed to the supervision and validation of the manuscript.

### Conflict of Interest

AZ received travel funding from Bayer-Schering and Sanofi Genzyme outside of the submitted work. ED'A received personal fees by Biogen and Sanofi. He also received travel funding from Bayer Biogen and Merck. GC received personal fees by Biogen and Sanofi. She also received travel funding from Bayer Biogen and Merck. GB received personal fees by Biogen and Sanofi. She also received travel funding from Bayer Biogen and Merck. CC has nothing to disclose LG and FP served on the advisory board for Bayer, Biogen Celgene, Merck, Novartis, Roche, Sanofi, Teva, and Almirall. They also received personal fees for speaking activities at congresses or sponsored symposia. The remaining authors declare that the research was conducted in the absence of any commercial or financial relationships that could be construed as a potential conflict of interest.
